# The functional therapeutic chemical classification system

**DOI:** 10.1093/bioinformatics/btt628

**Published:** 2013-10-30

**Authors:** Samuel Croset, John P. Overington, Dietrich Rebholz-Schuhmann

**Affiliations:** European Molecular Biology Laboratory, European Bioinformatics Institute (EMBL-EBI), Wellcome Trust Genome Campus, Hinxton, Cambridge CB10 1SD, United Kingdom

## Abstract

**Motivation:** Drug repositioning is the discovery of new indications for compounds that have already been approved and used in a clinical setting. Recently, some computational approaches have been suggested to unveil new opportunities in a systematic fashion, by taking into consideration gene expression signatures or chemical features for instance. We present here a novel method based on knowledge integration using semantic technologies, to capture the functional role of approved chemical compounds.

**Results:** In order to computationally generate repositioning hypotheses, we used the Web Ontology Language to formally define the semantics of over 20 000 terms with axioms to correctly denote various modes of action (MoA). Based on an integration of public data, we have automatically assigned over a thousand of approved drugs into these MoA categories. The resulting new resource is called the Functional Therapeutic Chemical Classification System and was further evaluated against the content of the traditional Anatomical Therapeutic Chemical Classification System. We illustrate how the new classification can be used to generate drug repurposing hypotheses, using Alzheimers disease as a use-case.

**Availability:** https://www.ebi.ac.uk/chembl/ftc; https://github.com/loopasam/ftc.

**Contact:** croset@ebi.ac.uk

**Supplementary information:** Supplementary data are available at *Bioinformatics* online.

## 1 MOTIVATION

Drug repurposing is the use of known active compounds for new therapeutic indications (Sanseau and Koehler, 2011). When administered in a living organism, a compound can indeed play various roles and affect different biological processes [called mode of action (MoA)]; accurately identifying these different functions helps to predict the potential side-effects a drug could have and can also lead to interesting repurposing opportunities (Medina-Franco *et al.*, 2008). For instance, ‘sildenafil’ was initially developed to relieve angina pectoris symptoms and has been repurposed towards erectile dysfunction during the clinical trials (Ashburn and Thor, 2004) when a new function of the target enzyme was discovered. Approved compounds are attractive because they have been extensively studied and have by definition already successfully passed clinical trials, where most drugs fail because of safety or efficacy issues. There is increasing number of approaches to predict repurposing opportunities using computational methods [see Dudley *et al.* (2011) or Andronis *et al.* (2011) for recent reviews]. Most methods operate on the profiles of physicochemical descriptors derived from molecular structures (Haupt and Schroeder, 2011). Other methods characterize the drugs on more abstract levels, such as the gene expression signature (Iorio *et al.*, 2010) or via the reported side-effects (Campillos *et al.*, 2008). These approaches have in common to look for similarities within existing drugs and forward similar compounds as repurposing hypotheses.

A feature of particular interest to describe drugs is the MoA. According to Wikipedia, the MoA describes ‘a functional or anatomical change, at the cellular level, resulting from the exposure of a living organism to a substance’. For instance terms such as ‘transcriptional regulation agent’ or ‘anticoagulant’ define MoAs and characterize the roles of a certain type of drugs. The MoA abstracts over the relations between molecular functions, protein targets and drug activities; it is the central concept linking a chemical structure to a set of biological activities. Intuitively, the indication of a drug logically depends on its MoA. Despite its widespread use in drug discovery, the MoA has not been used yet as a descriptor for repurposing analyses. One reason for this might be the challenge of formally defining MoAs. Indeed, MoAs are terms or categories, it is not possible to represent them straightforwardly with values and numbers like one can do for a 3D molecular structure or for a gene-expression profile. Nonetheless, the meaning of a concept can be formalized with controlled vocabularies and ontologies (Gruber, 1995); originating from description logics, such frameworks help to formalize the semantics of symbols and strings of characters with explicit axioms. In an ontology or knowledge base, ‘concepts’ (interchangeable with ‘category’, ‘term’ and ‘class’ in this article) are organized and linked following the logical type of relation they have among them. In the Gene Ontology (GO), for example (Ashburner *et al.*, 2000), biological processes and molecular functions terms are manually curated and their meaning specified by the relation types linking two GO terms. MoA definitions are present in other classifications such as the Medical Subject Headings (Nelson *et al.*, 2004) or the Chemical Entities of Biological Interest (Hastings *et al.*, 2013) for example. The Anatomical Therapeutic Chemical Classification System (ATC) (WHO, 2000) also describes to some extent the action of drugs at the anatomical level. All these resources are valuable for the community as a source of carefully and manually curated information. Moreover, the categories described in these classification systems are sometimes used to annotate drugs. For instance, the compound ‘sildenafil’ has been manually annotated as ‘vasodilator agent’ (CHEBI:35620 or MeSH:D27.505.954.411.918). The classifications mentioned previously are not specially designed for drug repurposing; they purposefully report only the well-known and major MoAs of chemical compounds. The pharmacological spectrum of each drug is not necessarily well covered, yet it would be the best way to predict new indications.

In our context, an ideal knowledge base would feature the known MoAs of a drug as well as some predicted ones to be tested in experiments. The MoA categories should derive and scale over primary molecular evidences exposed in biomedical databases, in an automated way. To address the lack of systematic MoA annotations, we have implemented the Functional Therapeutic Chemical Classification System (FTC), presented here in this article. The FTC is automatically built by leveraging the content of various biomedical databases using description logics and automated reasoning. Over 20 000 new MoA categories are defined in the resource and further populated with approved drugs using the Web Ontology Language (OWL) in combination with a reasoner. The population step takes in account the type of pharmacological action, the molecular targets of the drugs and their involvement into biological processes. Drugs can exhibit several MoAs, and the same MoA can be reached through different mechanisms. Most of the drugs are present in multiple FTC categories, reflecting the various roles a compound can play inside a biological system which can serve as starting point for repurposing. The resource was evaluated against the ATC, traditional classification scheme introduced before. We present as well some preliminary analyses over the data, by looking at the relation between the MoA and the indication of a compound using semantic similarity. Finally, we illustrate also how the FTC can be used as a pharmacological toolbox to analyze drug repurposing for Alzheimers disease.

## 2 RESULTS

**Fig. 1.**
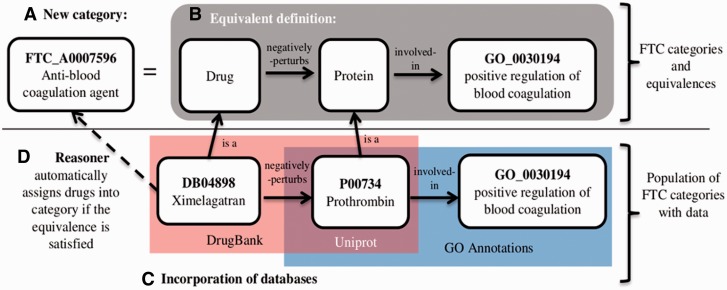
The diagram gives an overview of the integrated resources. (A) The name of FTC categories representing MoAs are directly derived from the GO terms representing the molecular functions and biological processes. A prefix ‘anti’ or ‘pro’ is appended to the original GO term, as well as the word ‘agent’ as suffix (e.g. the GO term ‘blood coagulation’ serves as a template to create the ‘anti-blood coagulation agent’ and ‘Pro-blood coagulation agent’ categories). (B) Each of the new FTC class has a logical equivalent definition assigned to it (axiom), representing the necessary and sufficient conditions for a drug to be classified in the corresponding MoA class. (C) The content of various databases is incorporated and linked using the FTC specific logical properties. (D) Finally a reasoner classifies the knowledge base and assigns drugs to MoA classes based on whether or not a definition can be satisfied. For example, the drug ‘ximelagatran’ will be assigned as member of the category ‘anti-blood coagulation agent’ because of the logical links ‘ximelagatran negatively-perturbs prothrombin’ and ‘prothrombin involved-in positive regulation of blood coagulation’. The taxonomic structure of the FTC appears also in the reasoning step, from the entailment of the equivalent definitions

The knowledge base behind the FTC is built by integrating information coming from various sources. The GO terms serve as template to create the FTC categories describing the MoAs; DrugBank (Knox *et al.*, 2011) provides the known links between drugs and their protein targets and Uniprot (The Uniprot Consortium, 2013) maps targets to their respective GO annotations (Dimmer *et al.*, 2012). Drugs are further assigned into MoA categories according to the OWL constructs and axioms defined in the FTC. A reasoner, a program capable of understanding such axioms, performed this task (see Section 5 for details). The process to build the FTC is summarized in Figure 1 alongside an example. The core step is the generation of axiomatic representations of MoAs by decomposing GO types into positive and negative regulations of biomolecular functions and processes. With the help of reasoning techniques, we can further derive and assign MoA across the knowledge base to given drugs. It requires a few seconds (four processing cores, 4-GB RAM) to classify the knowledge base [ELK reasoner, (Kazakov *et al.*, 2011)]. Other OWL reasoners (e.g. Hermit, Pellet, etc.) disqualified mainly due to long processing time (data not shown).

**Fig. 2.**
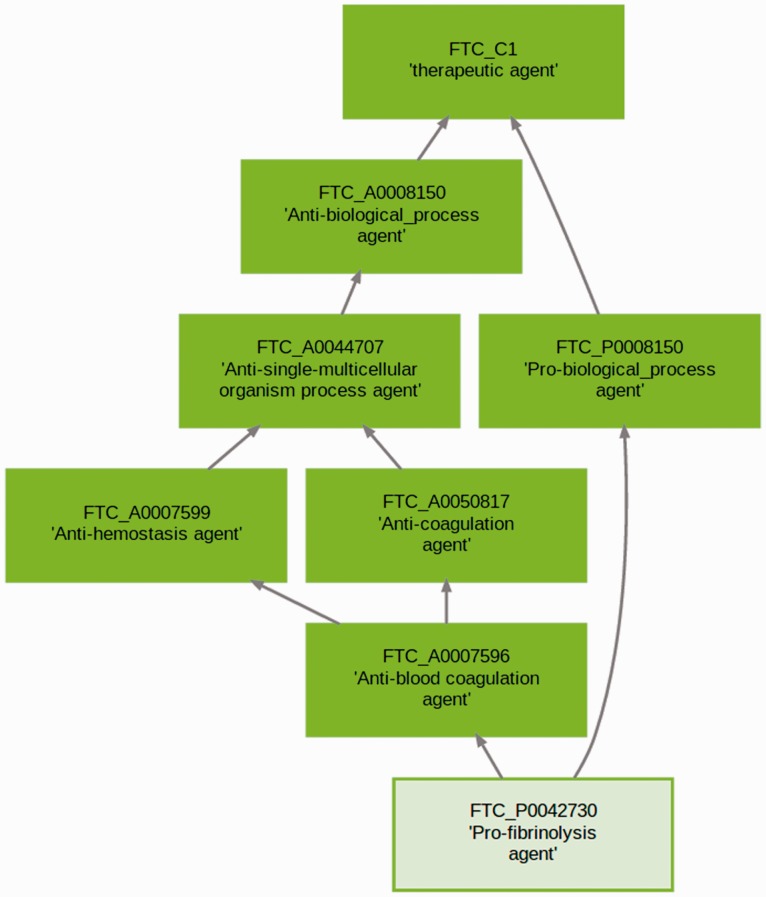
Parent categories to the FTC class ‘pro-fibrinolysis agent’ (FTC_P0042730). The classification is a direct acyclic graph where categories are describing increasingly specific concepts. Arrows entail subclass relationships between the terms (‘is a’ relation)

The FTC forms a taxonomic structure as illustrated on Figure 2, which arises when the reasoner classifies the knowledge base. In general, categories may have multiple parents and multiple children (see https://www.ebi.ac.uk/chembl/ftc for interactive use). In total there are 1280 FDA-approved DrugBank compounds (chemical and biotherapeutics) associated with 1264 human protein targets, where each drug is acting on at least one human protein target. The FTC introduces 23 353 new categories describing the mode and mechanism of action of therapeutic compounds. Of these categories, 4289 belong to the biological processes in GO and 19 064 to the molecular functions. A summary of the metrics behind the latest build is available online at https://www.ebi.ac.uk/chembl/ftc/evaluation/. Out of all FTC categories, 1432 categories (>6%) directly contain at least one approved drug. This number increases up to 2532 (>11%) when direct and indirect drugs are considered. FTC categories not containing drugs (e.g. FTC_A0001771 - Anti-immunological synapse formation agent) represent MoAs for which no approved compounds has qualified yet or that have not been identified as such in the FTC.

### 2.1 Evaluation

The content of the FTC has been evaluated against the drug categorization of the ATC, which has been produced by manual curation and serves as a gold standard. *A priori*, both resources serve different purposes and as a consequence, the evaluation has to take this into consideration (Section 3.2). The full methodology behind the evaluation is described in the Section 6 of the Supplementary Material.

Briefly, for 68 categories from the FTC, we can manually identify a set of semantically equivalent categories in the ATC. We call these equivalent categories the ‘evaluation points’ (see Supplementary Material Section 6.1 for details). All drugs from each evaluation point were then assessed to determine the quality of the FTC against our gold standard, i.e. the ATC. For example, the FTC category ‘Anti-hydrogen:potassium-exchanging ATPase activity agent’ (FTC_A0008900) has been manually asserted as equivalent to the ATC category ‘proton pump inhibitors’ (A02BC). A summary of this evaluation point is furthermore available online at https://www.ebi.ac.uk/chembl/ftc/evaluation/FTC_A0008900.

For 1280 DrugBank compounds in the FTC, 1134 are also present in the ATC, therefore only those were considered. The ‘evaluation points’ cover a total of 471 DrugBank compounds or around 41% of common drugs to both classifications. Out of these, 275 compounds are true positives, i.e. they match both, the FTC and ATC categories for a given evaluation point. The ‘proton pump inhibitor’ evaluation point is such a case where all the drugs (omeprazole, esomeprazole, pantoprazole, lansoprazole, rabeprazole) are present both in the FTC category and in the corresponding ATC category. The total number of compounds from an ATC category but where we could not identify a corresponding FTC category is 35 (false negatives). Finally, 280 compounds are present in a FTC class but not in any corresponding ATC category (false positives). Overall we derive a recall of 89%; this percentage indicates that the automatic build of the FTC covers a good portion of the content already present in the ATC. The precision of 50% shows that the FTC contains for a given MoA many more drugs than the equivalent ATC categories. This result was expected and comes from the original idea behind the FTC: Representing in a systematic fashion the implicit and explicit MoAs of drugs, in particular the ones not already indexed by current classification scheme.

### 2.2 Exploration

The FTC was designed to assist drug repositioning analyses by explicitly representing the polypharmacology of approved drugs. In this section, we exemplify how the resource can be used to perform different types of analysis.

#### 2.2.1 Polypharmacology spectrum

**Fig. 3.**
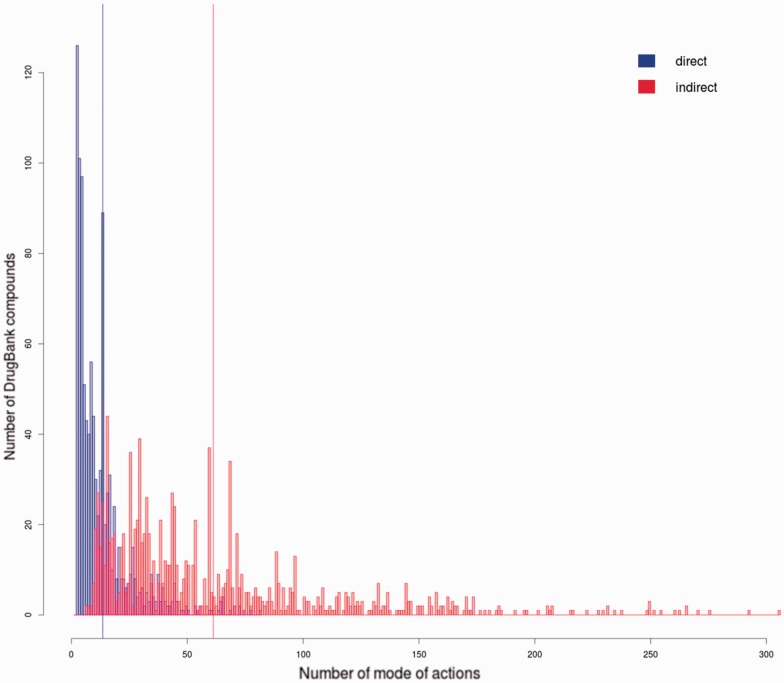
Distribution of the direct (blue) and indirect (red) number of MoAs per drug. Means are indicated with a solid line. On average each compound has 13.5 MoAs when only direct classes are considered. The number rises to 61.2 when indirect MoAs are included. Indirect MoAs are the ancestor classes in the taxonomy as shown in Figure 2. The distribution range is wider when indirect MoAs are considered (range = 299; min = 7; max = 306) versus direct MoAs only (range = 79; min = 3; max = 82). These results emphasize the fact that some drugs are well characterized in databases and could be used for a variety of specific biological tasks. Finally, some compounds have been assigned to a small number of FTC categories; in such cases little is known or reported about their pharmacology and repurposing opportunities might be limited

The more information on a drugs molecular targets and their physiological roles, the more opportunities exist to re-orient a drug into doing something new. The therapeutic agents described in the FTC can have several MoAs, i.e. may be acting on different biomolecular functions or processes, which demonstrates the intrinsic polypharmacology of the approved compounds. Figure 3 illustrates the polypharmacology spectrum by showing the distribution of number of MoAs per compound. When only direct categories are considered, compounds belong on average to 13.5 MoA categories. This number increases to 61.2 when parent categories are taken into consideration (super classes). Not all the MoAs are relevant to a disease, some FTC categories are particularly abstract (e.g. ‘Anti-biological process agent’) yet they represent discrete categories to which the drug belongs with an explicit and clear meaning. These discrete MoAs are a good starting point to understand what a compound can do when administered in a human system. Compound’s polypharmacology is well represented in the FTC, as shown by the numerous MoAs each approved drug can exhibit.

We decided to further look at a well-known repositioning example, in order to see whether the FTC was suitable to identify the new uses of an old drug. We picked the ‘thalidomide’ for this exercise (https://www.ebi.ac.uk/chembl/ftc/agent/DB01041). The molecule was first indicated to treat morning sickness in pregnant women, but has been quickly abandoned after its developmental toxicity has been discovered in newborns. The accepted molecular mechanism behind the side effect is an impairment of the angiogenic process responsible for the development of members, affecting in particular the limbs (Therapontos *et al.*, 2009). We found that the ‘thalidomide’ was accurately classified as ‘anti-cell migration involved in sprouting angiogenesis agent’ in the FTC, capturing the known toxicity of the drug. Furthermore, the ‘thalidomide’ is currently investigated for a multitude of new usages, in particular for anti-cancer and immunomodulatory activities among others (Teo *et al.*, 2005). These new indications are well represented in the FTC too, for example by the categories ‘anti-vascular endothelial growth factor production agent’ or ‘anti-cell division agent’ for antineoplastic activities, or by the classes ‘anti-cytokine secretion agent’ and ‘anti-I-kappaB kinase/NF-kappaB cascade agent’ for its effect on the immune system. These observations demonstrate that the FTC can successfully capture the molecular reasons behind the repositioning of an old compound. Moreover, the classification can also provide valuable insight regarding potential toxicity too.

#### 2.2.2 Drugs with similar MoAs have similar indications

The list of MoAs attributed to a drug can be exploited as a descriptor for the therapeutic agent: the tree structure of the FTC can be used to derive some similarity metrics over the MoAs. The underlying heuristic is to assume that the closer two entities are in the taxonomy, the more similar they are. We used a straightforward approach derived from the Jaccard index (see Supplementary Material Section 7) in order to compare approved drugs based on the similarity of their MoAs. For instance, the similarity between two compounds present in the same FTC category is 1 (maximum). The similarity between an ‘anti-blood coagulant’ and ‘pro-blood coagulant’ is 0.29, reflecting the fact that such compounds are dissimilar with regards to the outcome of their biological effect. As the MoA is intuitively expected to be the central concept leading to the indication of the drug, we expected that on average, drugs with similar MoAs would be indicated towards similar therapeutic areas.

**Fig. 4.**
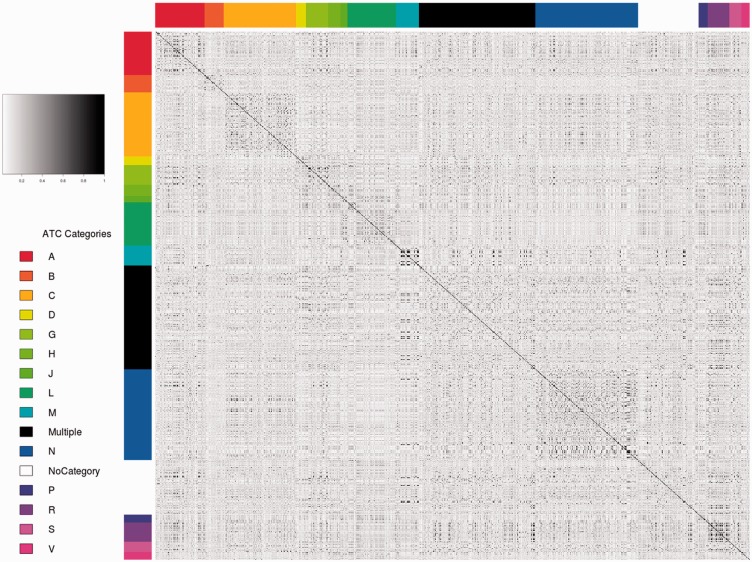
Pair-wise comparison of MoAs similarities. Therapeutic indications are represented by ATC categories which are the colors on the side. For instance, the compound ‘reteplase’ (DB00015) has the ATC code B01AD07, which appears as B (dark orange) on the plot. Only the first ATC level is considered. The reader can refer to Supplementary Figure S1A for a two ATC level granularity. The similarity descriptor ranges from 0 (not similar–white) to 1 (identical–black). Some compounds belong to multiple ATC categories (‘multiple’) and some others do not have an ATC code (‘NoCategory’). The average similarity of drugs present in the same therapeutic category is significantly higher on average when separately compared to all other indications

The heat map presented in Figure 4 shows a pair-wise comparison of all the drugs of the FTC based on their relative MoA similarity. The compounds are further grouped by therapeutic indications as defined by the ATC. The heat map reveals some square patches around the central diagonal; the overall similarity appears higher when compounds from the same ATC group are considered. A significance analysis (see Supplementart Material Section 8) revealed that the average MoA similarity of compounds belonging to the same ATC category is significantly higher than when compounds belonging to different categories are compared. Indeed, for each category, the *P*-value was inferior to 0.05 based on 20 000 random permutations over the similarity values. This result supports the idea that drugs with similar MoAs have similar indications. Note that the mean of the similarity values was considered for the statistical analysis; some outliers are also present in the map, which can be interpreted as repositioning hypotheses. These outliers have indeed similar MoAs, yet they belong to totally different therapeutic areas and are used for different purposes according to the ATC. We are currently further analyzing such cases in a systematic fashion. Hypotheses have to be manually examined and interpreted, as ATC categories are only covering some of the legal usage of the drugs. We expect to find off-label indications in the predictions for instance, as well as false positives.

Supplementary Figure S3 present similar association behavior when two levels of the ATC are considered (no statistical significance performed). Supplementary Figure S4 re-uses the same data as Figure 4 (one ATC level) but with a clustering function apply to it (hierarchical clustering-manhattan distance) in order to reveal functional clusters of drugs. Finally, Supplementary Figure S5 shows the distribution when compounds are sorted based on their identifiers; no patterns are identifiable in this case. Taken together, these results emphasize that the MoAs as defined in the FTC are indeed on average associated with the therapeutic indication of a drug. This result supports the validity of the resource and its potential to computationally address indication discovery.

### 2.3 Drug repurposing hypotheses for Alzheimer’s disease

**Fig. 5.**
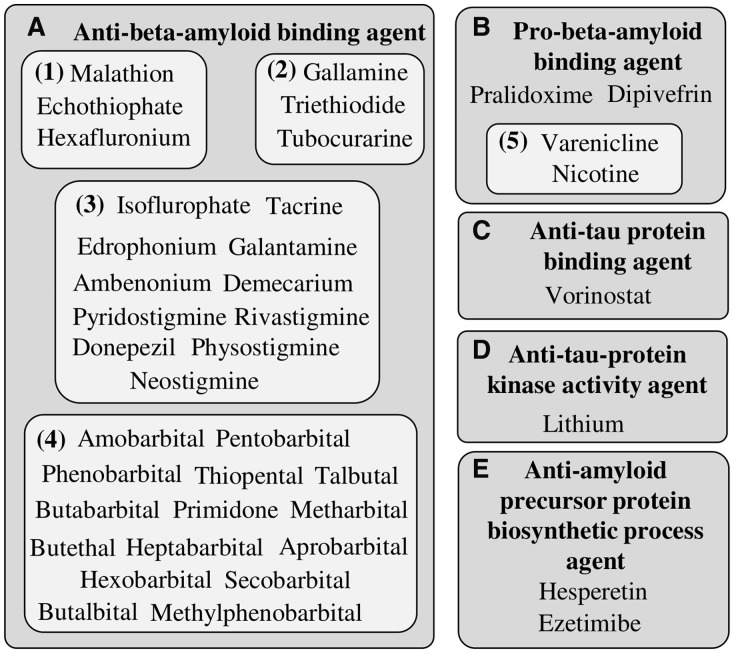
FTC categories describe some of the modes of action that could impact Alzheimer’s disease (letters on figure). The categories have been manually picked on the basis that they could directly affect the dementia. Drugs classified in these FTC categories further manually grouped based on their MoAs similarities (numbers on figure)

In this section, we provide examples how actual drug-repurposing hypotheses can be derived from the FTC. The approach presented here makes use of the FTC categories as analogs to compartments of a toolbox helping to find drugs to treat Alzheimer’s disease. Five FTC categories containing drugs are directly related to the biological processes of the neurodegenerative condition: ‘anti-amyloid precursor protein-biosynthetic-process agent’ (FTC_A0042983), ‘anti-Tau protein kinase-activity agent’ (FTC_A0050321), ‘anti-Tau protein binding agent’ (FTC_A0048156), ‘anti-beta amyloid binding agent’ (FTC_A0001540) and finally ‘pro-beta amyloid binding agent’ (FTC_P0001540). We have then considered the drugs present inside each of these classes as potential candidates. Figure 5 shows these drugs, which have been further manually grouped based on the overall similarity of their actions (numbers on Figure 5). The subgroups 1, 2 and 3 are inhibitors of the cholinergic system and some of them, such as ‘galantamine’ (DB00674), are already investigated to treat Alzheimer’s disease and other related dementias. This class of agent is in line with the cholinergic hypothesis (Francis *et al.*, 1999), stating that Alzheimer’s disease could be caused by dysfunctions in the processing of the acetylcholine. The subgroup 4 is exclusively composed of barbiturates (central nervous system depressants). The presence of this pharmacological class of compounds as an Alzheimer’s disease treatment is more surprising, as very little literature reports on it. Further investigations reveal that the neuronal acetylcholine receptor subunit alpha-7, a common off-target of barbiturates, binds beta-amyloids with high affinity (Wang *et al.*, 2000). As beta-amyloids are themselves strongly involved in the pathology, barbiturates could affect the state of the condition, similarly to cholinergic inhibitors (Wang *et al.*, 2000). The group B contains four compounds; ‘nicotine’ and ‘varenicline’ have been further grouped together because of similar pharmacology (group 5). ‘Nicotine’ has been shown to improve some of the symptoms of Alzheimer’s disease (Jones *et al.*, 1992), it is therefore expected to find this molecule in the predictions. ‘Varenicline’ possesses a pharmacology related to that of ‘nicotine’, which would explain the presence of the drug in this category. The two remaining drugs of group F are, respectively, ‘pralidoxime’ and ‘dipivefrin’; little information is available regarding their potential action against the condition, yet these compounds seem linked to the cholinergic hypothesis and could be considered for experimental testing. The groups C and D contain, respectively, one molecule each. These compounds have been classified as agents perturbing some of the physiological function of the Tau protein, key actor in Alzheimer’s disease (Grundke-Iqbal *et al.*, 1986). ‘Vorinostat’ (group C) is currently indicated for the treatment of cutaneous manifestations in patients with T-cell lymphoma, yet a study has shown *in vivo* (mouse model) the potential of the drug and other histone deacetylase inhibitors in regards to memory deficit (Kilgore *et al.*, 2010). The presence of the ‘lithium’ (group D) was confirmed by a recent study demonstrating a long-term protective effect for the ion in regards to Alzheimer’s disease (Young, 2011). The last group E contains ‘ezetimibe’ and ‘hesperetin’. These two compounds are primarily used as cholesterol lowering agents (statins). As cholesterol metabolism in the brain appears to be related to dementia, statins are believed to prevent or improve the symptoms of the patients. Although early studies (Wolozin, 2004) have failed to clearly show a beneficial effect, the investigation is still open. From the examples briefly presented above, reported and confirmed by the literature, the FTC appears to be suitable to identify real repurposing hypotheses tailored to a disease. Correctly identifying MoAs of interest helps to retrieve the compounds which might impact the treatment of a condition.

## 3 DISCUSSION

The FTC is a novel classification for approved drugs, which can be used as a starting point to generate drug repurposing hypotheses. This classification leverages the information present in various databases and ontologies, similarly to the Open PHACTS initiative (Williams *et al.*, 2012) and to the work done by Hoehndorf *et al.* (2012). The FTC mostly differentiates itself from these projects by providing a whole set of new categories on the top of the integrated information, dedicated to tackle a very specific problem: drug repositioning.

### 3.1 Biological assumptions

An asset of the FTC is its ability to handle efficiently categorical data: classes and relationships are accurately defined, in order to classify compounds based on the semantics of their relations. The properties linking drugs to their respective protein targets (‘positive’ and ‘negative perturbations’) are, however, simplistic. At the time being, no consideration is given regarding the binding strength between the drug and the proteins, yet it is a key factor to derive potent and specific activities in the human body. This is also the case for other types of numerical data, such as the dosage; the FTC can predict a role for a drug, yet it cannot provide any information about the concentration or the administration route necessary to obtain the potential effects. The current relations between targets and their involvement in biological processes are also not a fully accurate representation of the biological phenomenon. In a cell, specific domains of the protein could mediate different functions. Only one of such activity types can sometimes be inhibited by a drug (Kruger *et al.*, 2012), yet we are assuming in the FTC that as long as a drug affects a protein, it can therefore alter all its known functions. These limitations come from the semantics behind the axioms structuring the classification themselves based on the information available from the databases. Despite entailing not entirely accurately the biochemical reality, the axioms help to generate a larger number of hypotheses, the primary goal of the FTC. The dosage issue is partially addressed by the ‘regulator pattern’ (see Section 3.1 of Supplementary Material): it should be easier to experimentally adjust the concentration of the compounds classified as ‘pro-’ or ‘anti-’ biological process agents in order to modulate a physiological effect. The predictions generated by the FTC depend on the resolution of the curated information released by the original data providers. Erroneous or missing information will lead to misclassification by the reasoner. Some expected outcomes are also missing from the predictions; ‘sildenafil’ for instance was expected to be classified as ‘pro-penile erection agent’ (FTC_A0043084), yet the lack of appropriate GO annotation prevents it. After discussion with the GOA curation team, a manual annotation can only be asserted based on published experimental results. No document was found to support the involvement of the cGMP-specific 3’,5’-cyclic phosphodiesterase (sildenafil’s main target) in the ‘negative regulation of penile erection’ (GO:0060407), therefore no annotation can be made. Further work could be done in this direction, by trying to automatically infer more annotations or by using the electronically generated ones, in order to generate broader yet potentially less plausible repurposing hypotheses.

### 3.2 Interpreting the evaluation

Out of the evaluation, the high recall value (89%) supports the idea behind the automated build of the FTC: the data from different repositories funded and curated in parallel, can be integrated to automatically create a new resource. This new classification (FTC) contains most of the known information present in an external gold standard (ATC) and relies on description logics to leverage the native information. In the context of this work, we have compared the content of the FTC against the ATC, knowing that these two taxonomies have diverging goals. During the evaluation, equivalences have been manually asserted between classes, which are assumed to have fairly similar meaning and containing similar sets of compounds. These manual assertions are however a weakness, as they are themselves not evaluated (free parameter). The presence or absence of a link was determined only by one curator and any mistake can influence consequently the recall and precision values. The precision of 50% tells that the FTC tends to over-assigns compounds to MoA categories. The low precision value is acceptable in our case, as one of the underlying motivation of the FTC is to broadly represent polypharmacology, specially the one not present in gold standards such as the ATC, referencing only legal usage. In this regards, the evaluation should be considered more as a safety control rather than a formal assessment of a predictive method. The false positives derived from the evaluation can also be considered as drug repurposing hypotheses: these drugs can indeed be interpreted as suitable for the ATC category, yet not indexed as such. However, these predictions should be interpreted with caution, as it is currently impossible to distinguish a false positive from a reprofiling opportunity. These considerations do not interfere with the repurposing predictions generated based on semantic similarities or discrete categories as presented in the Section 2.2. Finally, note that the ATC/FTC equivalences are open and editable online, any modification will be automatically incorporated in the next release of the resource. It is also possible to evaluate the FTC against a different taxonomy, like the Medical Subject Headings for example, which can be subject to future work.

## 4 CONCLUSION

The FTC is public resource, which should assist drug repurposing initiatives or enhance computational studies that judge drugs according to their ‘mode of action’. The resource attributes biomolecular functions and processes to drugs, the same way as GO types have been assigned to gene products. The construction of FTC relies on axiomatic representations of MoA as the core means to attribute and derive the MoA for approved drugs. We shown the validity of the approach by comparing the content of the FTC to a well-established gold standard, the ATC. We further illustrate the tight relationship between the MoA and the indication of a drug and demonstrate using Alzheimer’s disease as an example how the resource helps to formulate drug repositioning hypotheses. The work leverages the semantics of distinct databases, working in parallel on different thematics. The platform will be further used to generate predictions in a systematic fashion, which can then be experimentally tested in the laboratory for validation.

## 5 AVAILABILITY AND IMPLEMENTATION

The code behind the creation of the resource is entirely open and available at https://github.com/loopasam/ftc. The web application and the FTC are built using the Brain library (Croset *et al.*, 2013) and can be find at https://www.ebi.ac.uk/chembl/ftc. The documentation can be accessed at https://github.com/loopasam/ftc/wiki. The full description of the methodology used to generate the classification is available as Supplementary Material.

## Supplementary Material

Supplementary Data

## References

[btt628-B1] Andronis C (2011). Literature mining, ontologies and information visualization for drug repurposing. Brief. Bioinform..

[btt628-B2] Ashburn TT, Thor KB (2004). Drug repositioning: identifying and developing new uses for existing drugs. Nat. Rev. Drug Disc..

[btt628-B3] Ashburner M (2000). Gene ontology: tool for the unification of biology. The Gene Ontology Consortium. Nat. Genet..

[btt628-B4] Campillos M (2008). Drug target identification using side-effect similarity. Science.

[btt628-B5] Croset S (2013). Brain: biomedical knowledge manipulation. Bioinformatics.

[btt628-B6] Dimmer EC (2012). The UniProt-GO Annotation database in 2011. Nucleic Acids Res..

[btt628-B7] Dudley JT (2011). Exploiting drug–disease relationships for computational drug repositioning. Brief. Bioinform..

[btt628-B8] Francis PT (1999). The cholinergic hypothesis of Alzheimer’s disease: a review of progress. J. Neurol., Neurosurg. Psych..

[btt628-B9] Gruber T (1995). Toward principles for the design of ontologies used for knowledge sharing?. Int. J. Human-Computer Stud..

[btt628-B10] Grundke-Iqbal I (1986). Abnormal phosphorylation of the microtubule-associated protein tau (tau) in Alzheimer cytoskeletal pathology. Proc. Natl Acad. Sci. USA.

[btt628-B11] Hastings J (2013). The ChEBI reference database and ontology for biologically relevant chemistry: enhancements for 2013. Nucleic Acids Res..

[btt628-B12] Haupt VJ, Schroeder M (2011). Old friends in new guise: repositioning of known drugs with structural bioinformatics. Brief. Bioinform..

[btt628-B13] Hoehndorf R (2012). Identifying aberrant pathways through integrated analysis of knowledge in pharmacogenomics. Bioinformatics.

[btt628-B14] Iorio F (2010). Discovery of drug mode of action and drug repositioning from transcriptional responses. Proc. Natl Acad. Sci. USA.

[btt628-B15] Jones GM (1992). Effects of acute subcutaneous nicotine on attention, information processing and short-term memory in Alzheimer’s disease.

[btt628-B16] Kazakov Y, Aroyo L (2011). Concurrent classification of Ontologies. The Semantic Web–ISWC 2011.

[btt628-B17] Kilgore M (2010). Inhibitors of class 1 histone deacetylases reverse contextual memory deficits in a mouse model of Alzheimer’s disease. Neuropsychopharmacol..

[btt628-B18] Knox C (2011). DrugBank 3.0: a comprehensive resource for ‘omics’ research on drugs. Nucleic Acids Res..

[btt628-B19] Kruger F (2012). Mapping small molecule binding data to structural domains. BMC Bioinformatics.

[btt628-B20] Medina-Franco JL (2008). Shifting from the single to the multitarget paradigm in drug discovery. Curr. Comput. Aided Drug Des..

[btt628-B21] Nelson SJ (2004). The MeSH translation maintenance system: structure, interface design, and implementation. Stud. Health Technol. Inform..

[btt628-B22] Sanseau P, Koehler J (2011). Editorial: computational methods for drug repurposing. Brief. Bioinform..

[btt628-B23] Teo SK (2005). Thalidomide as a novel therapeutic agent: new uses for an old product. Drug. Discov. Today.

[btt628-B24] The Uniprot Consortium (2013). Update on activities at the Universal Protein Resource (UniProt) in 2013. Nucleic Acids Res..

[btt628-B25] Therapontos C (2009). Thalidomide induces limb defects by preventing angiogenic outgrowth during early limb formation. Proc. Natl Acad. Sci..

[btt628-B26] Wang HY (2000). beta-Amyloid(1-42) binds to alpha7 nicotinic acetylcholine receptor with high affinity. Implications for Alzheimer’s disease pathology. J. Biol. Chem..

[btt628-B27] WHO (2000). Anatomical Therapeutic Chemical (atc) Classification Index with Defined Daily Doses (ddd’s).

[btt628-B28] Williams AJ (2012). Open phacts: semantic interoperability for drug discovery. Drug. Discov. Today.

[btt628-B29] Wolozin B (2004). Cholesterol, statins and dementia. Current Opinion in Lipidology.

[btt628-B30] Young AH (2011). More good news about the magic ion: lithium may prevent dementia. Br. J. Psychiatry.

