# Supplementary material for: The functional therapeutic chemical classification system
Source: Bioinformatics. 2013 Oct 30;30(6):876–83. doi: 10.1093/bioinformatics/btt628 (PMC3957075; doi:10.1093/bioinformatics/btt628)
Supplement: Supplementary Data [file supp_30_6_876__index.html]

The Functional Therapeutic Chemical Classification System — The functional therapeutic chemical classification system — The functional therapeutic chemical classification system — Supplementary Data 

# The functional therapeutic chemical classification system

## Supplementary Data

files

**Files in this Data Supplement:**

- Supplementary Data - pdf file
