# Supplementary material for: The functional therapeutic chemical classification system
Source: Bioinformatics. 2013 Oct 30;30(6):876–83. doi: 10.1093/bioinformatics/btt628 (PMC3957075; doi:10.1093/bioinformatics/btt628)
Supplement: Supplementary Data [file supp_btt628_revised-supplementary.pdf]

AVAILABILITY AND IMPLEMENTATION

This section describes the building mechanism behind the FTC. First, a list of *categories* describing the mode and mechanism of action of drugs is defined (the word *category* is interchangeable with *term*, *class* or *concept* in this manuscript). Then in a second step the newly created categories are automatically populated with approved compounds. Finally, the FTC is evaluated and repositioning hypotheses can be generated.

1 SOURCE CODE

The code behind the creation of the resource is entirely open and available at https://github.com/loopasam/ftc. The web application built on the top of the FTC can be found at https://www.ebi.ac.uk/chembl/ftc and the documentation can be accessed at https://github.com/loopasam/ftc/wiki. The reader should be familiar with description logics and the Web Ontology Language (OWL) to fully understand the construction of the knowledge base. An introduction to description logics from the perspective of the biomedical scientist is available on the wiki at https://github.com/loopasam/ftc/wiki/Description-Logics. We also refer the readers non-familiar with description logics and reasoning algorithms to excellent introductory text by Krötzsch *et al.* [2012]. The FTC implementation relies mostly on Brain [Croset *et al.*, 2013] and the web application builds on the top of the Play! framework [Play Framework, 2013]. Classification tasks use the ELK reasoner [Kazakov *et al.*, 2011]. The computer hosting the web application has 8 Gb of memory with 4 processors, this architecture allows fast parallel reasoning, thanks to ELK's design. More functionalities will be added to the web application following user requirements (so called *lean implementation*).

2 FTC CATEGORIES CREATION

The mode of action categories present in the FTC are defined based on the terms coming from the Gene Ontology (GO) [Ashburner *et al.*, 2000]. Both the molecular function and biological process branches are used for this purpose, yet handled slightly differently.

2.1 Categories related to biological processes

All the biological processes featured by the GO are looked-up one by one. All the time a process is linked to another process (*X*) via a *positive* or *negative regulation* link, two FTC classes are created: *Anti-X agent* and *Pro-X agent*. For instance the GO term *positive regulation of blood coagulation* is linked to the term *blood coagulation* via a *positively regulates* relation, therefore two FTC categories *Anti-blood coagulation agent* and *Pro-blood coagulation agent* are created. The identifiers of the new FTC classes are also derived from the GO term used to create the class pattern. The GO numeric identifier is re-used and the letter *A* or *P* is appended before to emphasize the *anti* or *pro* pattern. From the example presented previously, the FTC class *Anti-blood coagulation* has FTC_A0007596 as identifier, because the GO term *blood coagulation* is referenced by GO:0007596. Following the same logic, FTC_P0007596 is the identifier of the class *Pro-blood coagulation agent*. The design choice for identifiers and labels allows the FTC to fully rely on the high quality work provided by the GO curation team and scale over it.

2.2 Categories related to molecular functions

The mode and mechanism of actions related to molecular functions are created in the following manner: All the time a molecular function (*Y*) is encountered then two FTC categories are created, as for the processes: *Anti-Y agent* and *Pro-Y agent*. The identifiers are assigned the same way as described before. For instance, out of the GO term *androgen receptor activity* (GO:0004882) two FTC classes are derived: *Pro-androgen receptor activity agent* (FTC_P0004882) and *Anti-androgen receptor activity agent* (FTC_A0004882).

3 EQUIVALENT DEFINITIONS

FTC classes are generated as presented in the previous section. Up to this point, these categories are only tokens with a human readable label as well as an identifier. The next step is going to assign equivalent definitions to each FTC class. An OWL reasoner can understand such definitions and will automatically classify the knowledge base accordingly, following standard description logics reasoning services (see wiki for introduction). Drugs will then be assigned to FTC categories and the taxonomic structure arises after this reasoning step. Equivalent definitions are written as OWL class expressions using the entities of the knowledge base (summarised at https://github.com/loopasam/ftc/wiki/Knowledge-Base and in section 9). There are two types of equivalences: The first one captures perturbation of regulatory biological processes (so called *regulatory patterns*) and the second one handles the perturbed functions (*functional patterns*).

3.1 Regulatory pattern

Some of the FTC categories are created from the biological processes present in the GO (cf section 2.1); these categories have two arbitrary equivalent definitions, representing the two possible ways a compound might impact the biological process. Anti-biological process agent FTC categories contain the drugs that negatively perturb a target involved in the positive regulation of the biological process. The *anti* categories also feature the compounds that positively perturb a negative regulator of the same process. The *pro* categories are equivalent to the opposite pattern. Supplementary Figure 1 illustrates the equivalent definitions for the FTC class *Anti-blood coagulation agent* (FTC_A0007596).

3.2 Functional pattern

The FTC categories generated from the GO molecular functions (cf section 2.2) are also equivalent to a logical definition. *Anti* FTC categories dealing with molecular activities are asserted as equals to the drugs that negatively perturb the function. *Pro* categories are equivalent to the drugs that positively perturb the function of interest. A summary of the patterns definitions is available on the online wiki at https://github.com/loopasam/ftc/wiki/Mode-of-Action.

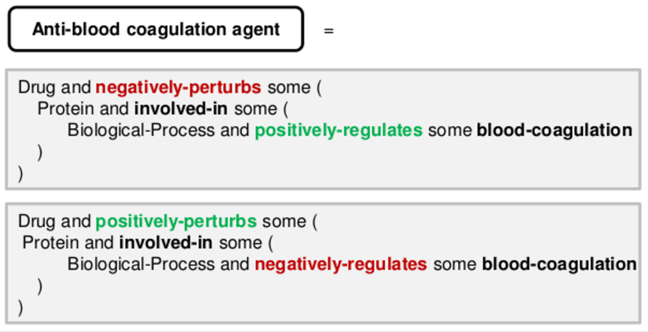

Supp. Fig. 1. Example of equivalent definitions (gray boxes) for the FTC category *Anti-blood coagulation agent* (FTC_A0007596). After the data integration step, a reasoner will look the definitions and identify which drugs satisfy the FTC definitions. Definitions are expressed using the Web Ontology Language (OWL), serialised here using the Manchester syntax.

4 DATA INTEGRATION

At this stage, the knowledge base contains the created FTC classes associated with their logical definitions, as well as the GO and the core FTC entities. The knowledge base is then further populated with some information coming from various public databases. Only manually curated information is considered.

4.1 DrugBank

The DrugBank database is a unique bioinformatics and cheminformatics resource that combines detailed drug data with comprehensive drug target information [Knox *et al.*, 2011]. The approved drugs (small molecules and biotherapeutics) acting on proteins are extracted from the database and imported in the FTC knowledge base. In order to be selected, a compound must firstly be approved and secondly have a pharmacological action on at least one human protein target present in Uniprot [The Uniprot Consortium, 2013]. The protein targets all have at least one manually asserted GO annotation [Dimmer *et al.*, 2012] for a biological process or a molecular function. DrugBank links compounds to targets via *actions*. The DrugBank actions are somehow structured and consistent: Concepts such as *inhibitor* or *agonist* are reused throughout the database for example, yet they are not strictly formalised as a controlled vocabulary. These actions are manually standardised to the core properties of the FTC according to their biochemical meaning: For instance the action *antagonist* is mapped to the FTC *negatively-perturbs* property. Compounds coming from DrugBank are represented as OWL classes and asserted as subclasses of the class *DrugBank compound* (FTC_C2). Protein targets are described as OWL classes too and subclasses of the core class *Protein*. Each DrugBank compound is then connected to its target via the following axiom pattern: *[drug] SubClassOf perturbs some [protein]*. E.g. *Ximelagatran SubClassOf negatively-perturbs some Prothrombin*.

4.2 Gene Ontology annotations

The GO annotation program aims to provide high-quality GO annotations to proteins in UniProt [Dimmer *et al.*, 2012]. In the context of the FTC, such annotations are used to create axioms linking protein targets to molecular functions and biological processes. Each protein annotated with a function creates an axiom such as *[protein] SubClassOf has-function some [molecular function]*. Each protein annotated to a biological process creates an axiom such as *[protein] SubClassOf involved-in some [biological process]*. E.g. *Prothrombin SubClassOf involved-in some positive regulation of blood coagulation*. Each protein can be involved in multiple processes and capable of realising multiple functions; some of the polypharmacology is captured at this level.

5 KNOWLEDGE BASE CLASSIFICATION

The knowledge base is fully built at this step and contains core classes, mode of actions descriptions alongside the actions of approved DrugBank compounds on protein targets in Uniprot. The proteins are linked to their molecular functions and involvement in biological processes via the GO annotations. The logical specifications of the FTC are there to glue the different data together and to explicitly express the logical links between resources. The FTC knowledge base follows an OWL2 EL profile [Motik *et al.*, 2009], which enable the use of fast and parallelised reasoners such as ELK. During the classification process, the reasoner checks whether the mode of action equivalent definitions are satisfied or not and assigns drugs inside the corresponding FTC categories. The tree structure of FTC appears also at this step from the logical definitions.

6 EVALUATION METHODOLOGY

As the classification of therapeutic agents is done in an automated way, it is important to evaluate the results generated against a known resource which will be considered as gold standard. The assessment of the FTC is done against another similar classification, the Anatomical Therapeutic Chemical Classification System (ATC) [WHO *et al.*, 2000]). The ATC has been developed to serve as a tool for drug utilization research in order to improve quality of drug use. In this resource, the information is manually curated, and drugs are assigned to categories based of their legally approved indications. The goal of the ATC differs from the one of the FTC, yet the two resources are sharing some very similar concepts, which can be used for the evaluation. Categories of both classifications contain approved drugs with a DrugBank identifier, meaning that some of the drugs indexed in the FTC are also present in the ATC. From that, it is possible to define some evaluation points, which will help to assess the automated classification process.

6.1 Evaluation Points

An evaluation point is defined as an equivalence between a class from the FTC with one or more classes from the ATC. The idea is to look at the set of drugs contained in both side of the equivalence and estimate the overlap, as illustrated in the Supplementary Figure 2. Evaluation points are defined by hand and not themselves evaluated. The full list of evaluation points as well as a summary of the results are available online at https://www.ebi.ac.uk/chembl/ftc/evaluation/. Each evaluation point has a series of true/false positive and false negative drugs associated with it.

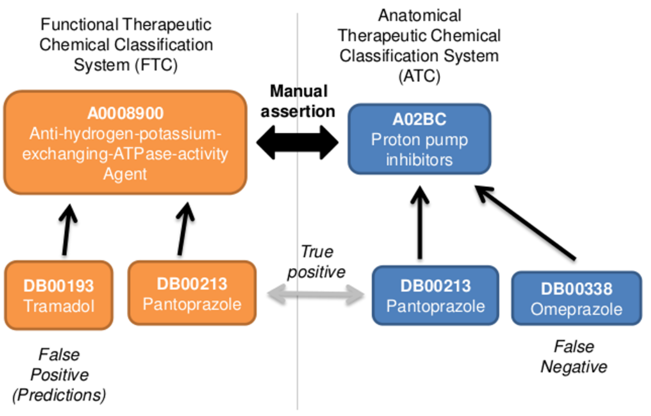

Supp. Fig. 2. Example of evaluation point. Classes from the ATC are manually mapped to classes from the FTC. The drugs present in each side of the equivalences are compared, and some metrics are derived from it (false positive/negative and true positive). A summary of the evaluation is available online at https://www.ebi.ac.uk/chembl/ftc/evaluation/.

6.2 True Positives

Drugs that are present in both the FTC and the equivalent ATC class(es) are called true positives. These compounds reflect that the automated classification was capable of retrieving correctly the information present in the gold standard (ATC).

6.3 False Negatives

These drugs are present in the ATC class(es) but not in the corresponding FTC class. The automated classification failed to retrieve these compounds. The smaller the number of false negatives is, the better the FTC is at recalling drugs. A small number of false negatives means that if a drug is present in the ATC (gold standard), then it is likely that the drug will also be correctly categorised in the FTC.

6.4 False Positives

The false positives are the drugs present in the FTC category of the evaluation point but not in the corresponding ATC classes. A high number of false positives means that the FTC is over-assigning compounds to classes. The false positives relates to the accuracy of the classification. In the context of this work, some false positives could also be considered as drug repurposing opportunities.

6.5 Precision

Precision is the probability that a randomly selected drug from the FTC is present in the equivalent ATC classes. The value is standardised as a percentage and corresponds to the formula: True Positive / (True Positive + False Positive).

6.6 Recall

Recall is the probability that a randomly selected drug from the ATC has been assigned to the correct corresponding class in the FTC. The value is standardised as a percentage and corresponds to the formula: True Positive / (True Positive + False Negative).

7 SEMANTIC SIMILARITY

The semantic similarity measure performed over the FTC is a derivative of the Jaccard index [Jaccard, 1912] [Rogers and Tanimoto, 1960] (see Pesquita *et al.* [2009] for review). It is probably best understood as an example: If we consider two classes A and B, the semantic similarity between these classes corresponds to the number of OWL superclasses (direct and indirect, obtained with a reasoner) that are shared by A and B (intersection) divided by the number of superclasses of A or B (union). The index ranges from 0 (totally different) to 1 (identical). A similar approach was successfully implemented by [Hoehndorf *et al.*, 2011], for similarity computations over phenotypic traits.

8 MODE OF ACTION SIMILARITY AGAINST INDICATION

A statistical analysis was performed over the data presented on Figure 4. When two compounds are randomly taken, they have on average a higher mode of action similarity when they are assigned to the same ATC category (one ATC level). In order to estimate whether this observation was due to chance only, we formulated the following null hypothesis (H0): *For a pair of drug A and B, it does not matter to which ATC category they belong to, their similarity is always average.* The alternative hypothesis (H1) was: *For a pair of drug A and B, if A and B have the same ATC code, we expect on average to obtain a higher similarity value than if A and B have different ATC codes.*

A permutation test was then performed for each ATC category. For example, we started with the ATC category A (first row on Figure 4), looked at the similarity values when pairs of compounds both belong to the category A (top right corner square) and compared it to the similarity values when the pair of compounds belong to different categories (A and B, A and C and so forth). For each comparison we obtained two distributions of values (not shown). On average the similarity values are always higher when the two compounds belong to the same category (A/A versus A/B for instance). A permutation test (n = 20’000) was performed in order to see whether this observation was due to chance only. We were able to reject the null hypothesis for a significance level of 0.05 all the times. The choice for a permutation test was driven by the fact that MoA similarity values do not follow any type of standard distribution (data not shown). For the significance levels set, the null hypothesis was rejected for all first level ATC categories.

9 KNOWLEDGE BASE SPECIFICATION

This section presents the scaffold of the knowledge base underlying the FTC. The logic structuring the FTC comes essentially from a set of core OWL properties (rich RBox). Some of these properties originate from the GO. When necessary some new ones have also been introduced. In order to understand how these properties interact, first will be presented the fundamental classes present at the top of the FTC classification.

9.1 Core classes

The high level concepts covered by the FTC are represented as OWL classes and enumerated below. Some of these core classes

are coming from external ontologies, in which case the original URI is preserved.

- **Molecular Function**
  Identifier: http://purl.obolibrary.org/obo/GO_0003674
  Label: molecular function
  Definition: As defined by the Gene Ontology. Elemental activities, such as catalysis or binding, describing the actions of a gene product at the molecular level. A given gene product may exhibit one or more molecular functions.

- **Biological Process**
  Identifier: http://purl.obolibrary.org/obo/GO_0008150
  Label: biological process
  Definition: As defined by the Gene Ontology: Any process specifically pertinent to the functioning of integrated living units: cells, tissues, organs, and organisms. A process is a collection of molecular events with a defined beginning and end.

- **Protein**
  Identifier: http://purl.uniprot.org/core/Protein
  Label: protein
  Definition: As defined by Uniprot. Description of a protein.
  Comment: Gene products present inside the FTC are all human proteins. Uniprot URIs are used.

- **Drug**
  Identifier: http://schema.org/Drug
  Label: drug
  Definition: As defined by schema.org: A chemical or biologic substance, used as a medical therapy, that has a physiological effect on an organism.
  Comment: In the context of the FTC, DrugBank chemicals are considered for their role as therapeutic agent rather than for their chemical structure. Mode of action classes are representing agent types and are subclasses of this concept.

- **Therapeutic Agent**
  Identifier: https://www.ebi.ac.uk/chembl/ftc/FTC_C1
  Label: therapeutic agent
  Definition: Role of a drug capable of producing a therapeutic effect.

- **DrugBank Compound**
  Identifier: https://www.ebi.ac.uk/chembl/ftc/FTC_C2
  Label: DrugBank compound
  Definition: Drug coming from DrugBank.

9.2 Core properties

The expressivity of the FTC comes mostly for the properties (RBox). Below is a list of the OWL object properties present inside the FTC knowledge base.

- **Part Of**
  Identifier: http://purl.obolibrary.org/obo/BFO_0000050
  Characteristic: Transitive
  Label: part-of
  Definition: As defined and used in the Gene Ontology.

- **Has Part**
  Identifier: http://purl.obolibrary.org/obo/BFO_0000051
  Characteristic: Transitive
  Label: has-part
  Definition: As defined and used in the Gene Ontology.

- **Regulates**
  Identifier: http://purl.obolibrary.org/obo/RO_0002211
  Chained property: regulates o part-of ->regulates
  Label: regulates
  Definition: As defined and used in the Gene Ontology.

- **Negatively Regulates**
  Identifier: http://purl.obolibrary.org/obo/RO_0002212
  SubPropertyOf: regulates
  Label: negatively-regulates
  Definition: As defined and used in the Gene Ontology.

- **Positively Regulates**
  Identifier: http://purl.obolibrary.org/obo/RO_0002213
  SubPropertyOf: regulates
  Label: positively-regulates
  Definition: As defined and used in the Gene Ontology.

- **Involved In**
  Identifier: https://www.ebi.ac.uk/chembl/ftc/FTC_R1
  Label: involved-in
  Domain: protein
  Range: biological process
  Definition: Entails the participation of a protein in a biological process.

- **Has Function**
  Identifier: https://www.ebi.ac.uk/chembl/ftc/FTC_R2
  Label: has-function
  Domain: protein
  Range: molecular function
  Definition: Describes the molecular function a protein can realize.

- **Perturbs**
  Identifier: https://www.ebi.ac.uk/chembl/ftc/FTC_R3
  Label: perturbs
  Domain: drug
  Range: protein
  Definition: Specific biochemical interaction through which a drug substance will affect the activity of a protein (mechanism of action). The property refers to the specific molecular targets to which the drug binds, such as an enzyme or receptor.

- **Negatively Perturbs**
  Identifier: https://www.ebi.ac.uk/chembl/ftc/FTC_R4
  Label: negatively-perturbs
  SubPropertyOf: perturbs

Definition: Specific biochemical interaction through which a drug substance will decrease the activity of a protein. The property refers to the specific molecular targets to which the drug binds, such as an enzyme or receptor.

- **Positively Perturbs**
  Identifier: https://www.ebi.ac.uk/chembl/ftc/FTC_R5
  Label: positively-perturbs
  SubPropertyOf: perturbs
  Definition: Specific biochemical interaction through which a drug substance will increase the activity of a protein. The property refers to the specific molecular targets to which the drug binds, such as an enzyme or receptor.

REFERENCES

Ashburner, M., Ball, C. A., Blake, J. A., Botstein, D., Butler, H., Cherry, J. M., Davis, A. P., Dolinski, K., Dwight, S. S., Eppig, J. T., Harris, M. A., Hill, D. P., Issel-Tarver, L., Kasarskis, A., Lewis, S., Matese, J. C., Richardson, J. E., Ringwald, M., Rubin, G. M., and Sherlock, G. (2000). Gene ontology: tool for the unification of biology. The Gene Ontology Consortium. *Nature Genetics*, **25**(1), 25–29.

Croset, S., Overington, J., and Rebholz-Schuhmann, D. (2013). Brain: Biomedical Knowledge Manipulation. *Bioinformatics Oxford England*, pages 1–2.

Dimmer, E. C., Huntley, R. P., Alam-Faruque, Y., Sawford, T., O'Donovan, C., Martin, M. J., Bely, B., Browne, P., Mun Chan, W., Eberhardt, R., Gardner, M., Laiho, K., Legge, D., Magrane, M., Pichler, K., Poggioli, D., Sehra, H., Auchincloss, A., Axelsen, K., Blatter, M.-C., Boutet, E., Braconi-Quintaje, S., Breuza, L., Bridge, A., Coudert, E., Estreicher, A., Famiglietti, L., Ferro-Rojas, S., Feuermann, M., Gos, A., Gruaz-Gumowski, N., Hinz, U., Hulo, C., James, J., Jimenez, S., Jungo, F., Keller, G., Lemercier, P., Lieberherr, D., Masson, P., Moinat, M., Pedruzzi, I., Poux, S., Rivoire, C., Roechert, B., Schneider, M., Stutz, A., Sundaram, S., Tognolli, M., Bougueleret, L., Argoud-Puy, G., Cusin, I., Duek-Roggli, P., Xenarios, I., and Apweiler, R. (2012). The UniProt-GO Annotation database in 2011. *Nucleic Acids Research*, **40**(Database issue), D565–70.

Hoehndorf, R., Schofield, P. N., and Gkoutos, G. V. (2011). Phenomenet: a whole-phenome approach to disease gene discovery. *Nucleic acids research*, **39**(18), e119–e119.

Jaccard, P. (1912). The distribution of the flora in the alpine zone. 1. *New phytologist*, **11**(2), 37–50.

Kazakov, Y., Krötzsch, M., and Simančík, F. (2011). Concurrent classification of Ontologies.

Knox, C., Law, V., Jewison, T., Liu, P., Ly, S., Frolkis, A., Pon, A., Banco, K., Mak, C., Neveu, V., Djoumbou, Y., Eisner, R., Guo, A. C., and Wishart, D. S. (2011). DrugBank 3.0: a comprehensive resource for 'omics' research on drugs. *Nucleic Acids Research*, **39**(Database issue), D1035–D1041.

Krötzsch, M., Simancik, F., and Horrocks, I. (2012). A description logic primer. *arXiv preprint arXiv:1201.4089*.

Motik, B., Grau, B. C., Horrocks, I., Wu, Z., Fokoue, A., and Lutz, C. (2009). OWL 2 Web Ontology Language Profiles. *Language*, **2009**(October), 1–53.

Pesquita, C., Faria, D., Falcao, A. O., Lord, P., and Couto, F. M. (2009). Semantic similarity in biomedical ontologies. *PLoS computational biology*, **5**(7), e1000443.

Play Framework (2013). http://www.playframework.com/.

Rogers, D. J. and Tanimoto, T. T. (1960). A computer program for classifying plants. *Science*, **132**(3434), 1115–1118.

The Uniprot Consortium (2013). Update on activities at the Universal Protein Resource (UniProt) in 2013. *Nucleic acids research*, **41**(Database issue), D43–7.

WHO *et al.* (2000). Anatomical therapeutic chemical (atc) classification index with defined daily doses (ddd's). *Oslo: WHO Collaborating Centre for Drug Statistics Methodology*.

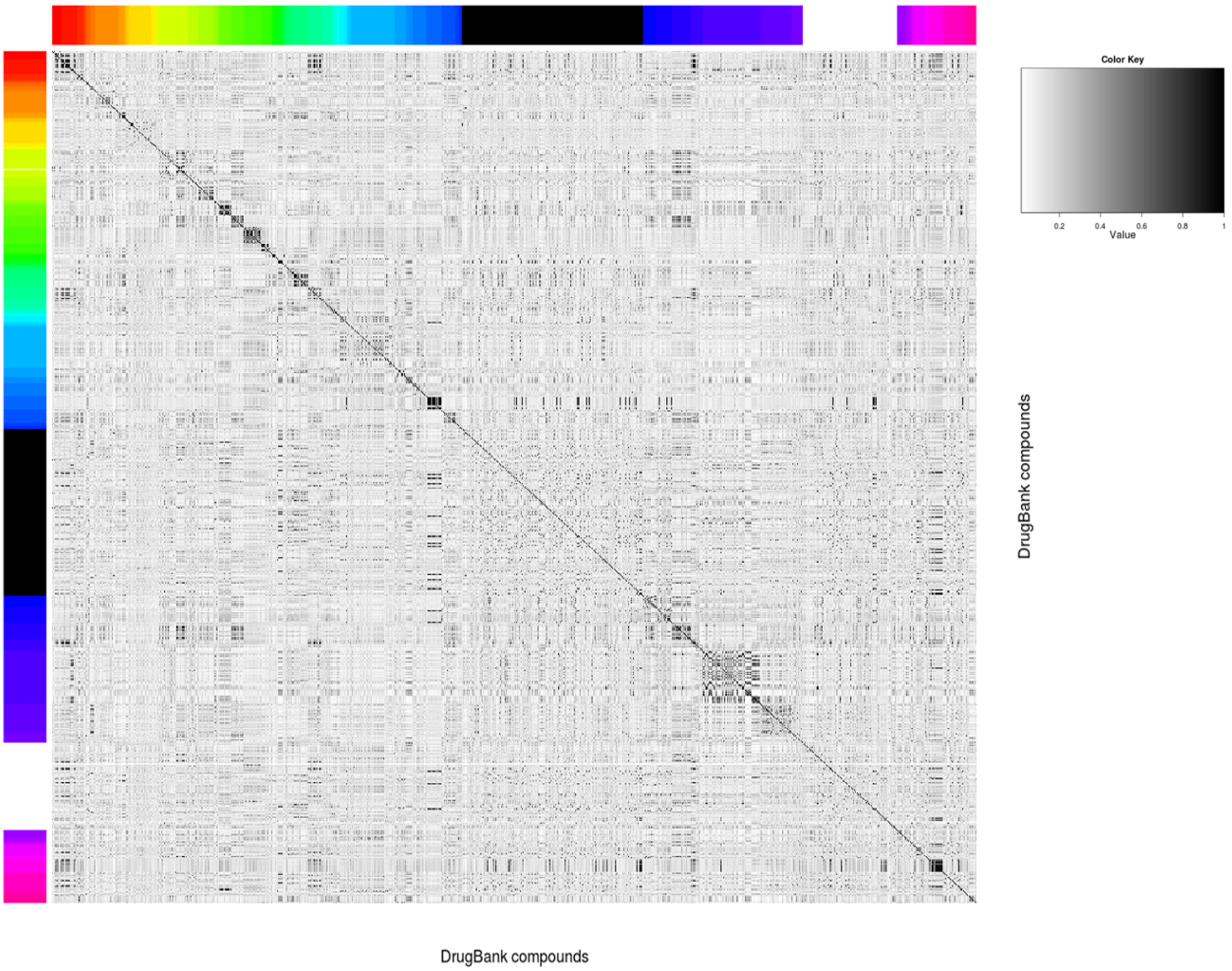

Supp. Fig. 3. Pairwise comparison of MoA similarities. This graph presents the same data as Figure 4 but with two ATC levels considered. It is possible to observe some clustering around the diagonal line (no statistical analysis performed).

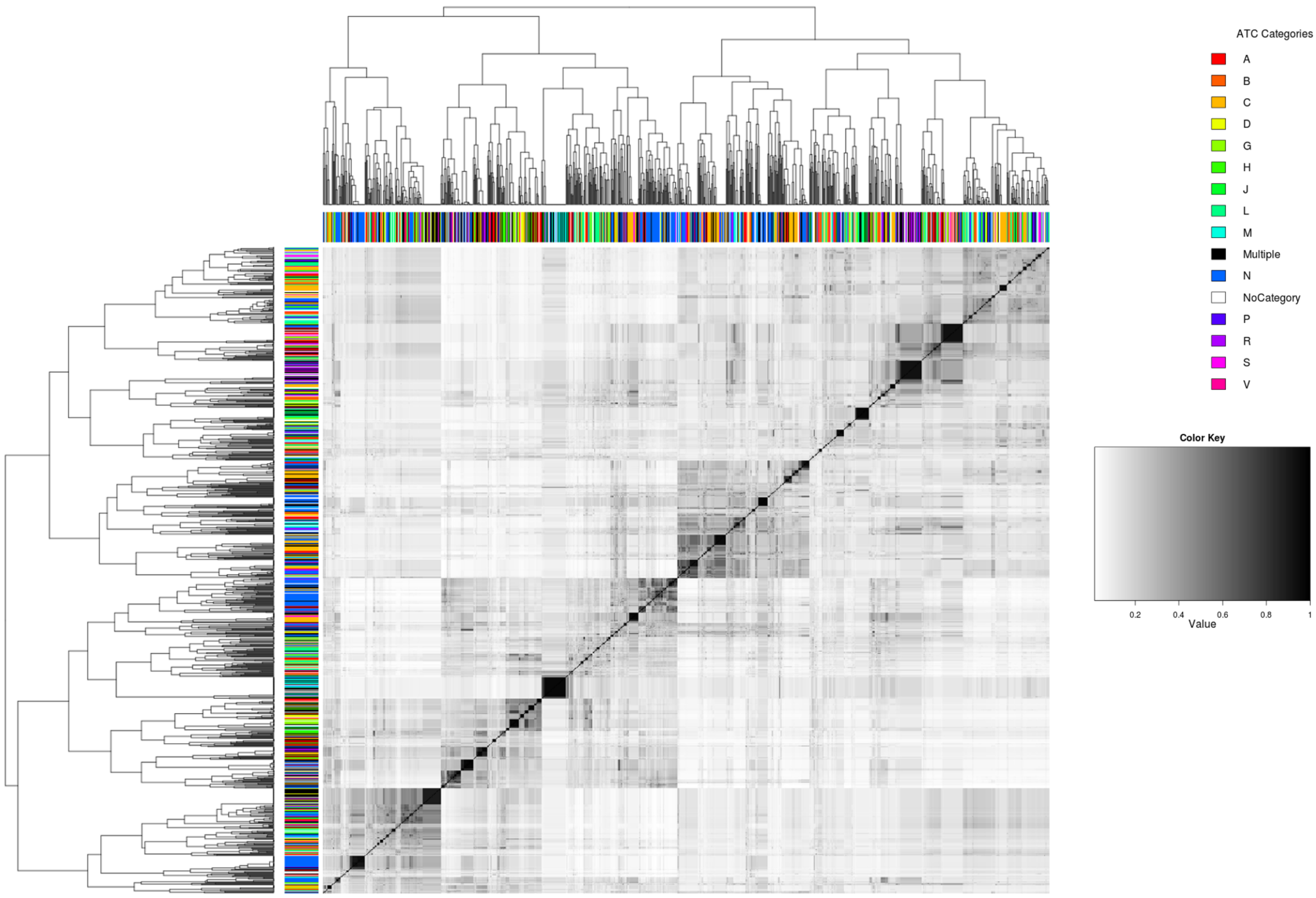

Supp. Fig. 4. Pairwise comparison of MoA similarities. This graph presents the same data as Figure 4 but with a hierarchical clustering applied on the top of it, based on the manhattan distance. The dendrogram reflects the original structure of the FTC: Functionaly similar compounds being grouped together. This figure can be used for further analysis over functional clusters of drugs.

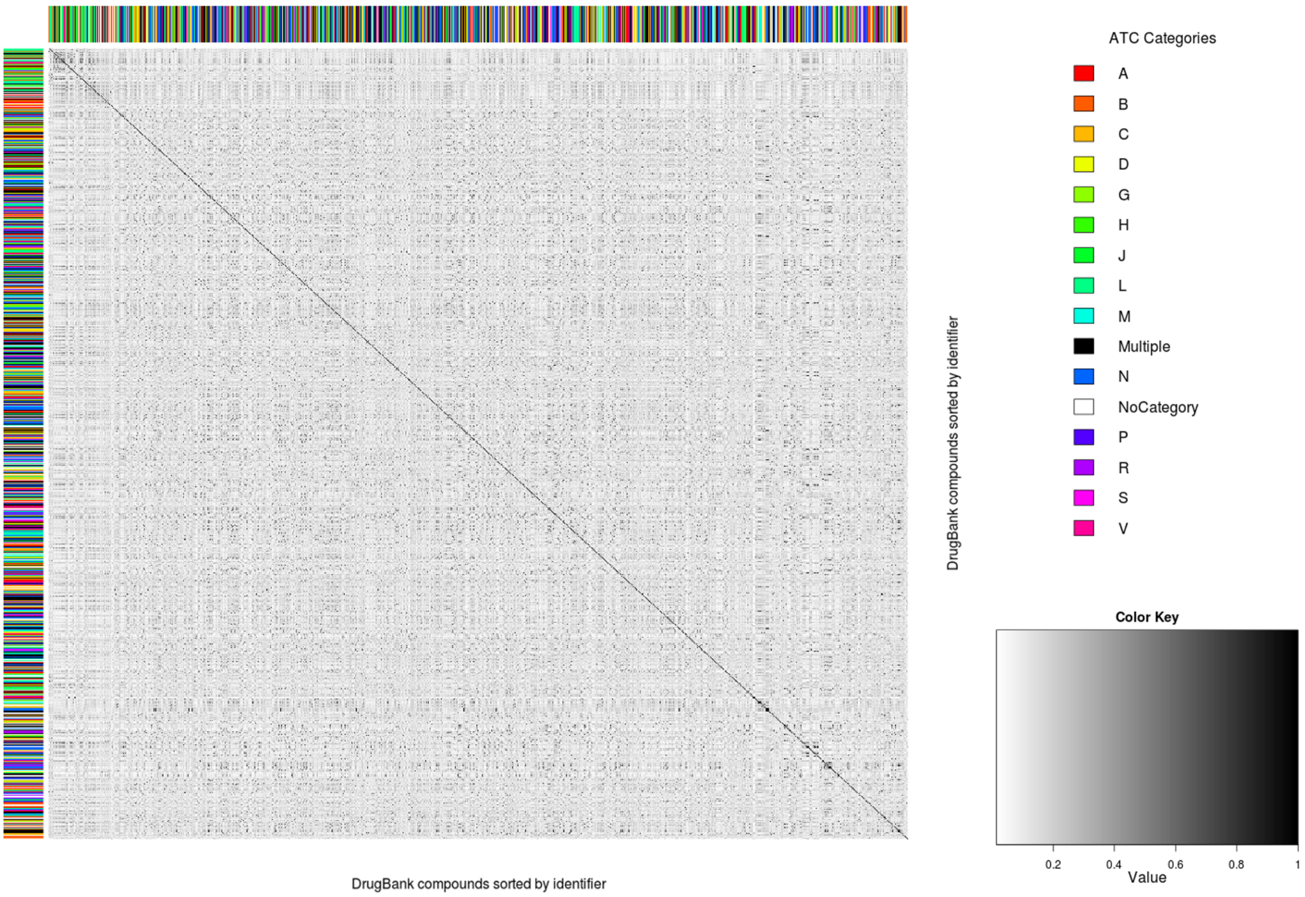

Supp. Fig. 5. Pairwise comparison of MoA similarities. This graph presents the same data as Figure 4 but the compounds have been sorted horizontally and vertically only based on their identifiers, which we considered as equivalent to a pseudo-random sorting. In this case no visual pattern is observable, the similarity values appear homogeneously distributed (no statistical analysis performed).
